# Visualizing the triheteromeric N-methyl-D-aspartate receptor subunit composition

**DOI:** 10.3389/fnsyn.2023.1156777

**Published:** 2023-05-24

**Authors:** Stephen Beesley, Akash Gunjan, Sanjay S. Kumar

**Affiliations:** Department of Biomedical Sciences, College of Medicine and Program in Neuroscience, Florida State University, Tallahassee, FL, United States

**Keywords:** NMDA receptors, subunit composition, *t*-NMDARs, visualizing subunit composition, immunohistochemistry, confocal microscopy, GluN3

## Abstract

N-methyl-D-aspartate receptors (NMDARs) are one of three ligand-gated ionotropic channels that transduce the effects of neurotransmitter glutamate at excitatory synapses within the central nervous system. Their ability to influx Ca^2+^ into cells, unlike mature AMPA or kainate receptors, implicates them in a variety of processes ranging from synaptic plasticity to cell death. Many of the receptor’s capabilities, including binding glutamate and regulating Ca^2+^ influx, have been attributed to their subunit composition, determined putatively using cell biology, electrophysiology and/or pharmacology. Here, we show that subunit composition of synaptic NMDARs can also be readily visualized in acute brain slices (rat) using highly specific antibodies directed against extracellular epitopes of the subunit proteins and high-resolution confocal microscopy. This has helped confirm the expression of triheteromeric *t*-NMDARs (containing GluN1, GluN2, and GluN3 subunits) at synapses for the first time and reconcile functional differences with diheteromeric *d*-NMDARs (containing GluN1 and GluN2 subunits) described previously. Even though structural information about individual receptors is still diffraction limited, fluorescently tagged receptor subunit puncta coalesce with precision at various magnifications and/or with the postsynaptic density (PSD-95) but not the presynaptic active zone marker Bassoon. These data are particularly relevant for identifying GluN3A-containing *t*-NMDARs that are highly Ca^2+^ permeable and whose expression at excitatory synapses renders neurons vulnerable to excitotoxicity and cell death. Imaging NMDAR subunit proteins at synapses not only offers firsthand insights into subunit composition to correlate function but may also help identify zones of vulnerability within brain structures underlying neurodegenerative diseases like Temporal Lobe Epilepsy.

## Introduction

N-methyl-D-aspartate receptors are remarkably functionally diverse– capable of modulating their kinetic and voltage-dependent properties for serving either as integrators or coincident detectors of synaptic activity to screening monovalent and divalent cations for regulating their selective permeabilities to bring about synaptic plasticity (Pilli and Kumar, 2014; Beesley et al., 2020b). Apart from glutamate, their endogenous ligand, they bind a host of molecules ranging from glycine and D-serine, their co-agonists, to ketamine, phencyclidine and zinc which modulate their function in ways still not fully understood. This diversity in function is attributed to unique assemblies of four subunit proteins that constitute the receptor’s subunit composition (Kumar, 2016). NMDAR subunit composition has hitherto been determined indirectly using cell biology, electrophysiology and/or pharmacology because imaging individual receptors or their subunits directly has proven difficult due to limitations in spatial resolution brought about by diffraction. This has hindered explorations into the role and locus of expression (presynaptic/postsynaptic) of the GluN3 subunit and its integration with GluN1 and GluN2 to make *t*-NMDARs in the brain. The recent availability of highly specific antibodies directed against extracellular epitopes of the subunit proteins has provided the opportunity for imaging their colocalization at synapses using high-resolution confocal microscopy as a means of examining subunit composition and testing specific hypotheses regarding their expression.

Glutamatergic NMDARs are heterotetrameric proteins comprising different combinations of the GluN1, GluN2 (A-D), and GluN3 (A-B) subunits derived from distinct gene families (*Grin1*-*Grin3*). All NMDARs contain one or more of the obligatory GluN1 subunits, which when assembled with GluN2 subunits of the same type, give rise to conventional diheteromeric (*d*-) NMDARs (e.g., GluN1-2A-1-2A). Note, however, that GluN3-containing *d*-NMDARs (e.g., GluN1-3A-1-3A), unlike their GluN2-containing counterparts, have been shown using expression systems to be activated by glycine but not glutamate, have reduced Ca^2+^ permeability, and believed to express presynaptically (Chatterton et al., 2002; Matsuda et al., 2002; Grand et al., 2018). Triheteromeric NMDARs, by contrast, contain three different types of subunits (e.g., GluN1-2A-1-2B), and include receptors that are composed of one or more subunits from each of the three gene families, designated *t*-NMDARs (Kumar, 2016) (e.g., GluN1-2A-3A-2A). We showed previously that GluN3-containing *t*-NMDARs in the brain can be distinguished from GluN2-containing *d*-NMDARs electrophysiologically, have reduced affinity for Mg^2+^ and increased selectivity for Ca^2+^ over Na^+^, making them highly Ca^2+^ permeable (Pilli and Kumar, 2012; Beesley et al., 2020b; Kumar and Kumar, 2021). These receptors are blocked by the pan-NMDAR antagonist D-(-)-2-Amino-5-phosphonopentanoic acid (D-AP5) and by D-serine, a potential gliotransmitter and a co-agonist of conventional NMDARs (Kumar, 2016; Beesley et al., 2019, 2020a). To obtain visual confirmation of the expression and colocalization of GluN1, GluN2, and GluN3 subunits to make *t*-NMDARs in native tissue, we immunoassayed individual subunit proteins in acutely cut slices of the rat brain (50 μm thick) with fluorescently tagged antibodies (Supplementary Table 1) and imaged them on a high-resolution confocal microscope. We looked specifically in the medial entorhinal area (MEA) where we had initially characterized the voltage-dependent properties of these receptors using electrophysiology, measured their Ca^2+^ permeability (Beesley et al., 2019, 2020b) and confirmed expression of the GluN3A protein and its colocalization with GluN1 and GluN2 (A and/or B) subunits (Kumar, 2016) using coimmunoprecipitation experiments (Beesley et al., 2019) and area specific tissue analysis (ASTA) (Beesley et al., 2022). Additionally, we determined whether these subunit proteins colocalized with PSD-95 or Bassoon, to determine the postsynaptic/presynaptic locus of their expression. The immunostained puncta imaged likely represent an ensemble of ∼10–20 NMDARs per synapse (Kumar and Huguenard, 2001; Goncalves et al., 2020; Li et al., 2021). Cross reactivity between different colored channels was minimized using appropriate secondary antibodies and fluorophores.

The premise of the current work is to determine whether: (a) NMDAR subunits can be individually visualized through immunohistochemistry in acute brain slices; (b) the subunit proteins co-express and overlap spatially to putatively inform about the subunit composition of the underlying receptors; (c) the subunits overlap with Bassoon and/or PSD-95 to inform about their pre- and/or postsynaptic colocalization. This type of imaging is qualitative in nature and precludes any meaningful quantitation because the images acquired pertain to only a single optical section from a stack of confocal-acquired images (collapsing the stack makes the puncta difficult to resolve because their size in the z-plane is at most the size of the minimum optical thickness possible on the scope). Furthermore, differences in the antigenicity of the fluorophore-conjugated antibodies required adjustment of intensity levels for each of the fluorophores imaged to effectively declutter and resolve the individual puncta. Thus, in addition to minimizing cross immunofluorescence of the fluorophore-conjugated antibodies, we had to optimize imaging using the luminescence/contrast correction parameters for each of the channels separately such that only the brightest puncta for each fluorophore could be visualized. This may jeopardize the accurate counting/estimation of subunit puncta and/or synapses on dendrites in the regions imaged, and hence the goal of this study is restricted to establishing/confirming whether subunit proteins for assembling *t*-NMDARs are expressed by the brain and determining the locus of their expression. Quantitation of expression levels is therefore beyond the scope of the current work and may require more sophisticated approaches like FRET (fluorescence resonance energy transfer) imaging and/or electron microscopy.

## Materials and methods

All experiments were carried out in accordance with the *National Institutes of Health Guide for Care and Use of Laboratory Animals* and were approved by the Florida State University Institutional Animal Care Committee. Although no experiments were conducted on live animals, we have followed the recommendations in the ARRIVE guidelines.

### Brain fixation and slicing

As described previously (Beesley et al., 2020a,^2022^), Sprague-Dawley rats (male, postnatal day 40–90, 160–190 g, *N* = 4) were deeply anesthetized with urethane (1.5 mg/kg; i.p.) prior to intra-aortal fixation with 4% paraformaldehyde (PFA) in a 0.1 M phosphate buffer solution (PB; pH 7.4; 4^°^C) following an initial flush with ice-cold saline (0.9%, 4^°^C). Brains were removed and post-fixed overnight in PFA before being transferred to a 30% sucrose solution in PB until equilibration. Horizontal slices (50 μm-thick) were cut on a cryostat and the sections (six series comprising of 12 sections per series) collected in a cryoprotectant solution consisting of 30% ethylene glycol and 25% glycerol in 50 mM PB. The cut sections were stored at –20^°^C until processed or analyzed.

### Imaging

#### Immunofluorescence

Cryo-protected brain slices fixed in PFA were trimmed to retain the regions of interest (MEA and hippocampus) and washed in PB (0.1 M; 2, 5 min rinses), main rinse solution (MRS: 0.1 M PB, 0.1M glycine, 0.5% Triton X-100; 3, 10 min rinses) before being exposed to a blocking solution (0.1 M PB, 0.5% Triton X-100, 2% goat serum, 2% bovine serum albumin) for a minimum of 1 h on a shaker. Slices were then exposed to the primary antibody (Supplementary Table 1) in blocking solution overnight at room temperature under agitation. Slices were then washed in PB (3, 5 min rinses), MRS (3, 10 min rinses) before being exposed to the secondary antibodies (Supplementary Table 2) in blocking solution for 2 h under agitation. For multi-antigen immunolabeling, primary and secondary antibodies from differing host species were generally incubated together e.g., GluN1 (*guinea pig*) and GluN2A (*rabbit*). However, as many of the primary antibodies used were raised in rabbit, we did sequential primary-secondary antibody incubations with intermittent exposures to an unconjugated goat anti-rabbit secondary antibody to saturate as many epitopes on the primary as possible. For example, to assay for GluN2A and GluN3A subunit proteins in rat with primary antibodies made in rabbit, we first incubated the tissue with rabbit anti-GluN1 primary antibody overnight, washed with PB and MRS before exposing it to goat anti-rabbit Alexa-488 secondary antibody for 2 h. Following this step and washes with PB and MRS, the tissue was incubated with an unconjugated goat anti-rabbit secondary antibody for 2 h before being washed again in PB and MRS and exposed to the second rabbit anti-GluN3A primary overnight. Finally, following PB and MRS washes, the tissue was incubated with the third goat anti-rabbit Alexa-594 secondary antibody for 2 h before being rewashed in PB and MRS and readied for mounting on glass slides using vectashield mounting media with or without DAPI (Vector Laboratories, CA, USA). This sequential incubation protocol enabled successful labeling of multiple antigens despite the limitation of finding primary antibodies made in different host species. MAP2 protein was immunolabeled by incubating slices overnight in a rabbit primary (Supplementary Table 1) in blocking solution. They were washed in MRS (3, 10 min rinses) the following day and incubated in goat anti-rabbit biotin for 2 h and streptavidin 647 (all in blocking solution; Supplementary Table 2) for 2 h with intermittent washes in MRS (3, 10 min rinses). Each immunofluorescence assay was repeated at least twice with brain sections taken from different series.

**FIGURE 4:**
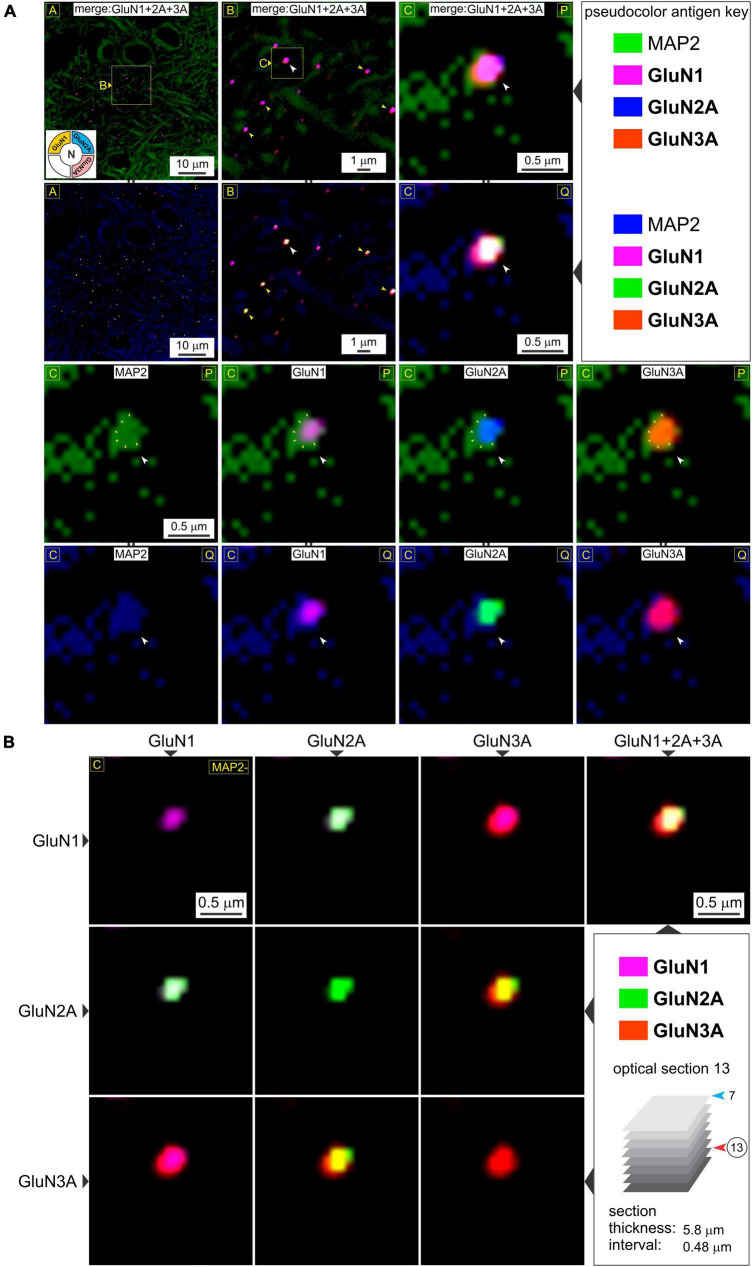
A closer look at *t*-NMDAR subunit composition. **(A)** Consistency in immunolabeling of colocalized GluN1, GluN2A, and GluN3A subunit protein puncta (*merged images* in R1-2C1-3) relative to dendrites (MAP) in deeper potions of the medial entorhinal area (MEA) [optical section 13 versus 7 in previous figure; *see bottom right panel* in **(B)**] at the indicated enlargements (*lettered boxes in yellow*). Arrowheads (in images at the top two rows) point to representative examples of subunit colocalization and changes in the *pseudocolor antigen key* (marked by || between images; R2C1-3, R4C1-4) are to aid in gauging colocalization of the proteins imaged. An example of a putative spine head emanating from the dendrite at high magnification (*images* in R1-2C3, R3-4C1-4) containing a postsynaptic density (PSD) (outlined by the small yellow arrowheads in images R3C1-4) where the individual subunits (white arrowheads, R3-4C1-4) appear to coalesce. **(B)** The *merge matrix* for pairwise assessment of GluN1, GluN2A, and GluN3A subunit protein colocalization at a single synapse at the highest level of magnification. The merged image in the rightmost column (R1C4) showcases *t*-NMDAR subunit composition (Note differences in *pseudocolor antigen key* with Figure 3B).

The glass mounted slides were stored at 4^°^C in the dark until imaged on a confocal laser-scanning microscope (Zeiss LSM 880) using a Plan-Apochromat 63x/1.40 oil DIC M27 objective with appropriate excitation/emission filters for the secondary antibodies listed in Supplementary Table 2. During initial microscope setup (line averaging: 4; pixel dwell: 2.67–5.33 μs; resolution: assigned by software) the fluorophore with the longest wavelength imaged in an experiment (e.g., 647 nm) was assigned 1 Airy unit and the pinhole size corresponding to this setting (e.g., 64 μm) was used for each of the other fluorophores imaged under high magnification. Laser intensity and optical gain was set based on the fluorophores imaged such that the energy used for excitation saturated as few of the pixels as possible. The acquired images were minimally processed using Zen 2012 SP1 (black edition; Carl Zeiss) software where we took advantage of the digital zoom (with interpolation) to enlarge them, assign channel colors to the fluorophores, and optimize intensity and contrast (using the range indicator function) so that only the brightest signals/puncta could be visualized without background clutter. These likely represent protein agglomerations on spine heads that are at just the right orientation in the optical plane to maximize viewing. We used the program’s built-in (Min/Max and Best Fit) functions to guide us in optimizing the imaging of each channel separately using the luminescence/contrast correction parameters (Gamma/Black/White) as exemplified in Supplementary Figure 6. Colocalization/overlap of protein puncta was ascertained using these independently optimized images at high/low digital zooms and depicted in figure form throughout the manuscript for various experimental conditions and controls. Furthermore, we make no distinctions between object and pixel based colocalization given the high magnification used for visualization of the subunits and do not construe colocalization alone to be indicative of protein-protein interactions. Unless indicated otherwise, images used in the figures are from a single optical section in a z-stack of ∼10–15 sections per brain slice (section thickness: ∼5.8 μm; interval 0.48 μm; see Figure 4B).

### Cell culture and transfection

Human embryonic kidney (HEK) 293 cells were grown to 80% confluence in six-well plates in DMEM (Dulbecco’s Modified Eagle Medium) containing fetal bovine serum at 37^°^C and 5% CO_2_ and transfected with fluorescent protein-tagged GluN subunit-specific plasmids using jetPRIME transfection reagent (Polyplus) as per the manufacturer’s instructions. The following plasmids were used for transfections: GluN1, pEYFP-NR1a (Addgene plasmid # 17928) (Luo et al., 2002); GluN2A, pCI-EGFP-NR2a wt (Addgene, plasmid # 45445) (Barria and Malinow, 2002); GluN3A, pcDNA3.1-NR3A-eGFP, which we constructed for this study as follows: Plasmid pGEMHE-NR3A-eGFP designed for *in vitro* transcription was a generous gift of Dr. Ehud Isacoff, University of California at Berkely (Ulbrich and Isacoff, 2008). The NR3A-EGFP fusion gene from this plasmid was excised using BamH1 and *Not*I restriction enzymes and ligated in frame into the corresponding restriction sites in the multiple cloning site of the mammalian expression vector pcDNA3.1 V5-A to generate the pcDNA3.1-NR3A-eGFP plasmid. After verifying the cloning junctions and part of the coding sequence by Sanger sequencing, this plasmid was used in our study for HEK 293 cell transfections. A total of 72 h post-transfection, cells were washed once in phosphate buffered saline and lysed in 20 μl 1x SDS sample buffer (in mM): 50 Tris (pH 6.8), 10% glycerol, 2.5 EDTA (pH 8), 2% SDS, 2.5% β-mercaptoethanol, 2 mg bromophenol blue, boiled for 5 min at 100^°^C and centrifuged for 1 min at 16,000 g.

### Cell Biology

#### Immunoblotting

For assaying NMDAR subunits, a 6% polyacrylamide gel was used. Total HEK cell protein was added to the gel and allowed to migrate in running buffer (in mM): 25 Tris, 191 glycine, 0.1% SDS at 180V for approximately 75 min, or until the dye front ran off the gel. The gel was then transferred to polyvinylidene difluoride (PVDF) membrane using a transfer buffer (in mM): 25 Tris, 191 glycine at 75V for 90 min at 4^°^C. The membrane was then blocked for 30 min in 5% fat-free milk in Tris-buffered saline (TBS) and incubated with the primary antibody (Supplementary Table 1) overnight at room temperature under gentle agitation. Following removal of the primary antibody on the following day, the membrane was washed in TBS with Tween-20 (TBST; in mM: 50 Tris, 150 NaCl pH 7.4–7.6, 0.05% Tween-20; 3, 5 min rinses) and incubated with the secondary antibody [donkey anti-rabbit IRDye 680RD or donkey anti-guinea pig IRDye 680RD (LI-COR)] for 1 h in the dark at room temperature under gentle agitation. Membranes were rewashed in TBST (3, 5 min rinses) and then imaged on a LiCor Odyssey CLx imager. To rule out cross reactivity, primary antibodies (anti-GluN3A, anti-GluN1 and anti-GluN2A) were added sequentially to the membrane and intermingled with separate imaging sessions i.e., anti-GluN3A, donkey anti-rabbit IRDye 680RD exposure → imaging session #1 → anti-GluN1, anti-guinea pig IRDye 680RD exposure → imaging session #2 → anti-GluN2a, donkey anti-rabbit IRDye 680RD → imaging session #3 (see Supplementary Table 1).

### Statistical tests

Unless otherwise noted, statistical significance was measured with a nested *t*-test (GraphPad Prism 9). Error bars in the figures represent standard error of the mean.

### Data availability

All data generated and/or analyzed during this study are included in this published article. Note that the low magnification images in the figures represent *raw data* used for analysis of proteins imaged under enlargement of regions identified in these images. However, TIFF versions of the images used and/or analyzed during the current study are available from the corresponding author on reasonable request.

## Results

### GluN1 and GluN3 subunit proteins colocalize with PSD-95 on dendrites

**FIGURE 1:**
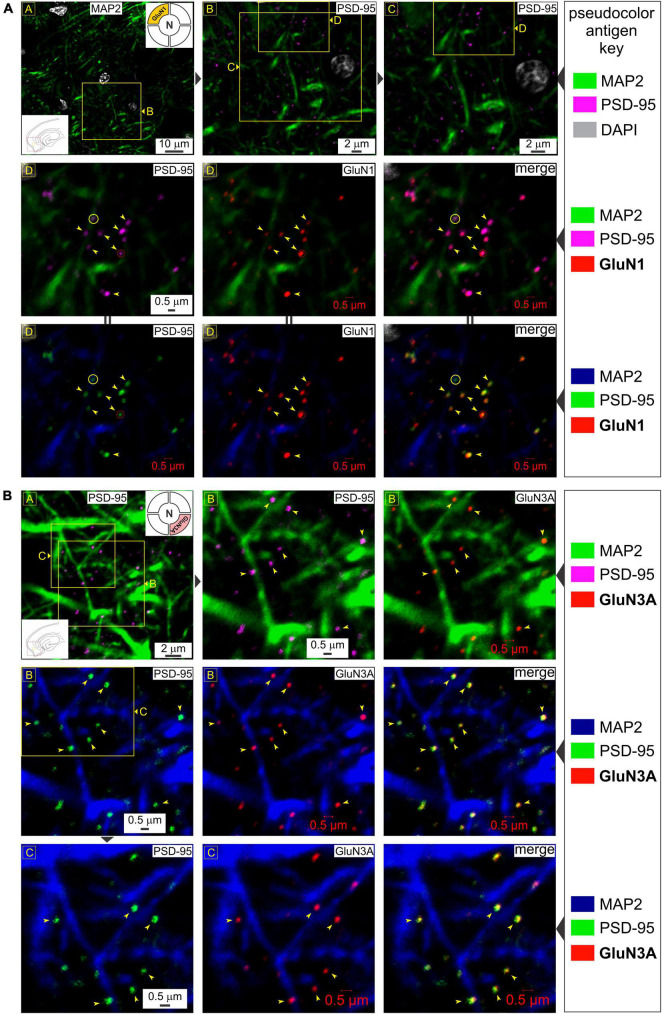
Glycine-binding GluN1 and GluN3A subunit proteins colocalize with the postsynaptic marker PSD-95. **(A)** *Top row*: Immunolabeling of postsynaptic density (PSD-95) puncta in the medial entorhinal area [MEA, also referred to as *area entorhinalis pars medialis* in the literature; red box in the bottom left *inset* of image in R (*row*) 1, C (*column*) 1] relative to dendrites immunolabeled with MAP2 at the indicated enlargements (lettered boxes in yellow; images magnified successively are linked with ▶). Nuclei are labeled with DAPI. In this and subsequent figures, the *pseudocolor antigen key* indicates the color assignment for the antigens/fluorophores imaged/depicted and alterations in color assignment (marked by || between images) are to aid in gauging colocalization of the proteins imaged. *Middle and bottom rows*: Immunolabeling of GluN1 subunit protein of the NMDAR (N; top right *inset* of image in R1C1) and their colocalization (*arrowheads*) with PSD-95 (*merged images* in R2C3 and R3C3) at putative synapses on dendrites (*red circles*). Note that not all PSDs contain GluN1 (*yellow circles*). **(B)** Immunolabeling of PSD-95 puncta (images in R1C1, R1C2, R2C1, and R3C1) and GluN3A (top right *inset* in R1C1) subunit protein (images in R1C3, R2C2, and R3C2) and their colocalization (arrowheads) with PSD-95 (*merged images* in R2C3 and R3C3) at the indicated enlargements (lettered boxes in yellow). Note that the schematics of brain slices embedded as insets within images and keys in this and all subsequent figures, provide locational information of the regions imaged (*red boxes*) and approximately where within these regions the images depicted are taken from (*yellow boxes*). We have been consistent in recording from the same location within the MEA, and hence, some insets convey just region-specific information.

To determine synaptic versus extrasynaptic expression of the critical glycine-binding subunits of *t*-NMDARs (GluN1 and GluN3) in the MEA, we immunoassayed neuron-specific dendrites with microtubule-associated protein 2 (MAP2), postsynaptic density with PSD-95 protein which is exclusively localized to mature glutamatergic synapses (Prange et al., 2004; Zheng et al., 2011), and either GluN1 (Figure 1A) or GluN3A (Figure 1B). The dendritic marker enables delineation from the soma (identified with the nuclear stain DAPI, or 4′,6-diamidino-2-phenylindole*, top row*, Figure 1A) and lends perspective to the relative location of putative synapses through PSD-95 immunolabeling. Note the punctate expression of the postsynaptic marker relative to the dendrite at various magnifications (*top row*, Figure 1A) and the precision with which the GluN1 protein puncta tend to coalesce (*arrowheads*, *middle row*, Figure 1A). Not all PSDs appeared to contain GluN1 (*circled yellow*, *middle row*, Figure 1A), suggestive of nascent synapses (Washbourne et al., 2002). Alterations in the pseudo color antigen key for the proteins imaged facilitate the gauging of their colocalization (*middle and bottom rows*, Figure 1A). Note the preponderance of GluN3A protein puncta, which like GluN1, colocalize nicely with PSD-95 (arrowheads, *all rows*, Figure 1B). Together, these data show the synaptic expression of both GluN1 and GluN3A proteins in the MEA through their colocalization with postsynaptic marker PSD-95.

**FIGURE 2:**
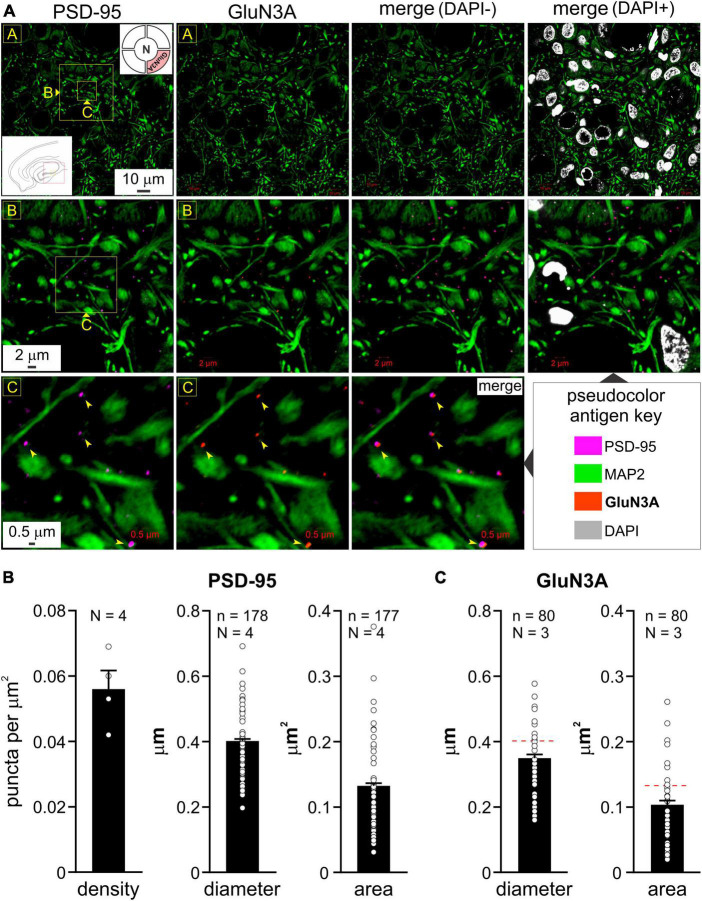
Colocalization of GluN3A subunit protein with postsynaptic density (PSD-95) in the hippocampus and quantitation of puncta in the medial entorhinal area (MEA). **(A)** Immunolabeling of PSD-95 puncta (images in R1C1, R2C1, and R3C1) and GluN3A (top right *inset* in R1C1) subunit protein (images in R1C2, R2C2, and R3C2) and their colocalization with PSD-95 (*merged images* in R1C3-C4, R2C3-C4, and R3C3; *arrowheads*) in the hippocampus (CA1-3; red box in the bottom left *inset* of image in R1C1) relative to dendrites immunolabeled with MAP2 at the indicated enlargements (*lettered boxes in yellow*). Nuclei, labeled with DAPI, indicate cell density within the neuropil. **(B,C)** Histograms of average density, diameter, and area of PSD-95 **(B)** and GluN3A **(C)** puncta in the MEA (error bars indicate s.e.m; n, number of puncta measured; N, animals used). Density estimates **(B)** were only made for PSD-95 from various non-overlapping regions within MEA imaged at differing magnifications. GluN3A subunit protein puncta were on average smaller in diameter and cross-sectional area compared with PSD-95 [hatched red line, **(C)**], although these differences were not statistically significant, *p* = 0.21 and 0.24, respectively, nested *t*-test.

To validate our imaging data in the MEA, we also assayed the hippocampus where GluN3A expression was established using area specific tissue analysis (ASTA) (Beesley et al., 2022). Consistent with these studies, we found widespread expression of GluN3A protein puncta that colocalized with PSD-95 throughout CA1 to CA3 (Figure 2A), although the high magnification used for their visualization precluded quantification of their relative abundance in these subfields. Note the dendritic enmeshing of cell bodies in what appears to be *stratum pyramidale* (*top row*, Figure 2A) and the coalescing of GluN3A puncta with PSD-95 at the level of the dendrites in the neuropil at various magnifications (*bottom rows*, *arrowheads*, Figure 2A). The average PSD-95 density in the MEA was 0.056 ± 0.006 puncta per μm^2^ (mean ± s.e.m, *N* = 4 animals; Figure 2B) and the average diameter (0.402 ± 0.006 μm) and cross-sectional area (0.131 ± 0.004 μm^2^) of these puncta (*n* = 178 and 177, respectively, Figure 2B) were similar to those reported in the literature (Sheng and Hoogenraad, 2007; Kim and Sheng, 2009). Interestingly, GluN3A subunit protein puncta were on average smaller in diameter and cross-sectional area (0.350 ± 0.011 μm and 0.104 ± 0.006 μm^2^, respectively, *n* = 80, *N* = 3; Figure 2C) compared with PSD-95 (Figure 2B), although these differences turned out to be not statistically significant (*p* = 0.21 and 0.24, respectively, nested *t*-test). Despite minor variations in size due to spine orientation etc., the consistency of the averaged data with electron microscopy studies reported in the literature lend confidence to our imagining the colocalization of distinct NMDAR subunits at a single synapse.

### GluN1, GluN2, and GluN3 subunit proteins colocalize postsynaptically for making *t*-NMDARs

**FIGURE 3:**
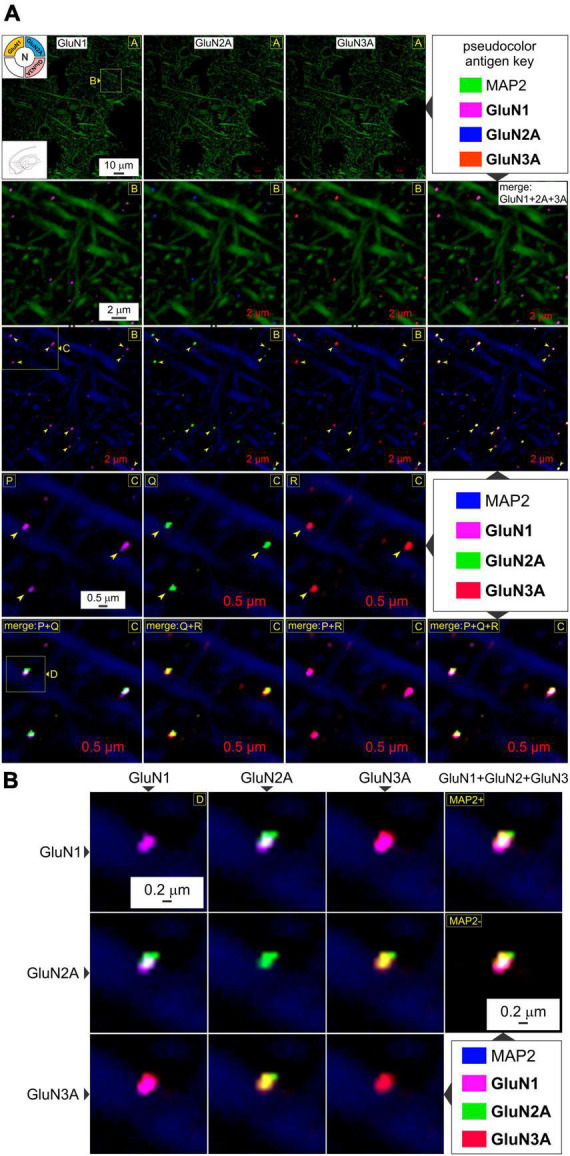
GluN1, GluN2A, and GluN3A subunit proteins colocalize for making *t*-NMDARs in the MEA. **(A)** Quadruple immunolabeling of GluN1 (images in R1-4C1), GluN2A (images in R1-4C2), and GluN3A (images in R1-4C3) subunit protein puncta relative to dendrites immunolabeled with MAP2 in the MEA (red box in the bottom left *inset* of image in R1C1) and their pairwise and triplicate colocalization (*merged images* in R2-3C4, R5C1-4) for making *t*-NMDARs (top left *inset* in R1C1) at the indicated enlargements (*lettered boxes in yellow*). Arrowheads point to representative examples of individual subunit puncta or their colocalization. Changes in the *pseudocolor antigen key* (marked by || between images) are to aid in gauging colocalization of the proteins imaged. **(B)** The *merge matrix* for pairwise assessment of GluN1, GluN2A, and GluN3A subunit protein colocalization at a single synapse on a dendrite at the highest level of magnification. The merged images in the rightmost column (R1-2C4) showcase *t*-NMDAR subunit composition, comprising glycine binding subunits (GluN1 and GluN3A) coalescing with glutamate binding subunits (GluN2A).

**FIGURE 5:**
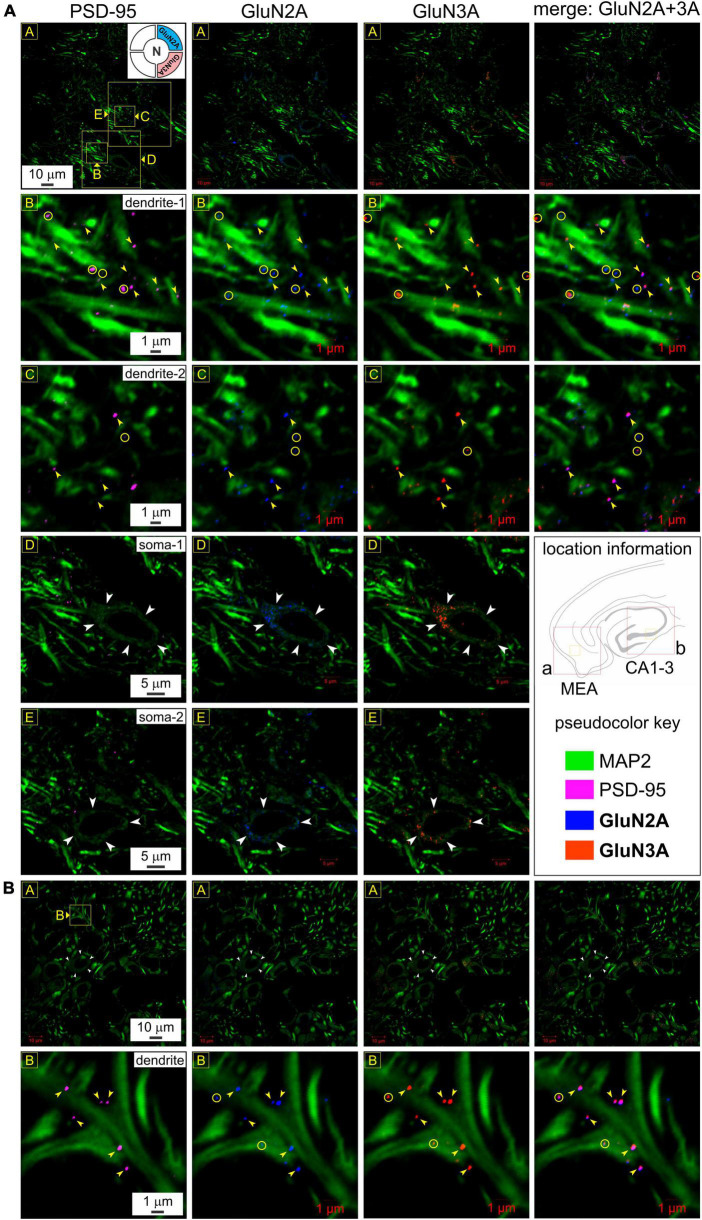
Postsynaptic expression and colocalization of glutamate binding (GluN2A) and glycine binding (GluN3A) subunits in medial entorhinal area (MEA) and hippocampus. **(A)** Immunolabeling of GluN2A and GluN3A subunit proteins and their colocalization with postsynaptic density (PSD-95) to reconfirm their postsynaptic expression in MEA **(A)** and the hippocampus **(B)** at various levels of enlargement (*lettered boxes in yellow*). GluN3A and GluN2A puncta colocalize with PSD-95 at the level of the dendrites (MAP2; R2-3C1-4), as can be seen by correlating the merged images of GluN2A and GluN3A (*rightmost column* of images, R1-3C4) with PSD-95 (*leftmost column* of images, R1-3C1) at the indicated magnifications. Yellow arrowheads point to representative examples of individual subunit puncta, their colocalization with each other and with PSD-95. Note that not all subunits colocalize with PSD-95 or with each other (*yellow circles*). Dense immunolabeling of GluN2A and GluN3A subunit proteins, but not PSD-95, in the perikaryon of the cell bodies (*white arrowheads*, R4-5C1-3), but not the nucleus (internal control for colocalization of subunits with the postsynaptic marker). **(B)** Somatic (*white arrowheads*, R1C1-4) and dendritic (*yellow arrowheads*, R2C1-4) immunolabeling of GluN2A and GluN3A subunit proteins in the hippocampus (CA1-3) follow the pattern observed in MEA **(A)**. Note how the coalesced subunits (R1-2C4) align with the postsynaptic marker (R1-2C1) at the level of the dendrite under high magnification (yellow arrowheads, *bottom row*). Not all colocalization could be associated with a visible PSD (yellow circles, *bottom row*).

**FIGURE 6:**
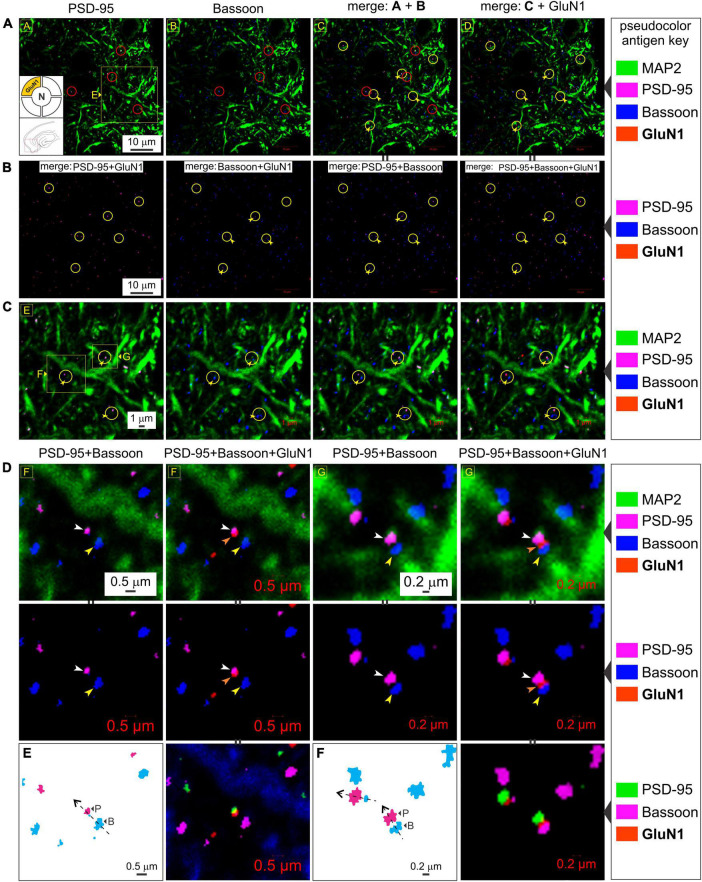
The GluN1 subunit protein colocalizes with postsynaptic density (PSD-95) but not the presynaptic marker Bassoon. **(A–C)** Quadruple immunolabeling of GluN1 (mandatory subunit of all NMDARs), PSD-95 (postsynaptic marker), Bassoon (presynaptic marker) and MAP2 (dendrite) in the medial entorhinal area (MEA) highlighting the separation between pre- and postsynaptic markers (*red circles* in a) and/or GluN1 [*yellow circles with embedded arrowheads* in **(A–C)**] and the dendritic colocalization of GluN1 subunit protein with PSD-95 but not Bassoon [rightmost columns in **(A–C)**] at the indicated enlargements (*lettered boxes in yellow*). Note the distinction between colocalization of GluN1 puncta with PSD-95, and together, their pairing with a clearly separated Bassoon. Changes in the *pseudocolor antigen key* [marked by || between images in **(A,B)**] are to aid in gauging colocalization of the proteins imaged. **(D–F)** Expression of GluN1 subunit protein puncta (*orange arrowheads*) relative to PSD-95 (*white arrowheads*) and Bassoon (*yellow arrowheads*) at single synapses under high magnifications **(D,E)**. The *pseudocolor antigen key* is altered to aid gauging of colocalization of the proteins imaged [marked by || between images in **(D–F)**]. The GluN1 puncta outlined in **(E,F)** relative to PSD-95 (P) and Bassoon (B) is indicative of synaptic orientation (*dotted lines with arrows*) in the optical plane imaged.

**FIGURE 7:**
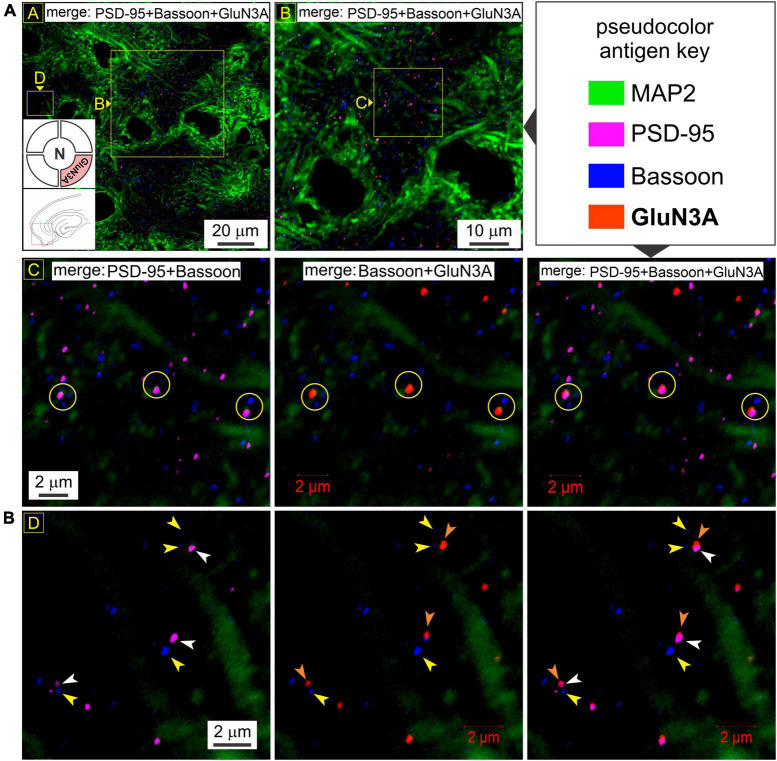
GluN3A subunit protein also colocalizes with postsynaptic density (PSD-95) but not Bassoon. **(A)** Quadruple immunolabeling of GluN3A (required for making *t*-NMDARs), PSD-95 (postsynaptic marker), Bassoon (presynaptic marker) and MAP2 (dendrite) in the medial entorhinal area (MEA) showing the separation between pre- and postsynaptic markers (R2C1) and/or GluN3A (top two rows; *yellow circles* in R2C1-3) and the dendritic colocalization of GluN3A subunit protein with PSD-95 but not Bassoon (*merged images* in R1C1-2 and R2C2-3) at the indicated enlargements (*lettered boxes in yellow*). **(B)** Dendritic expression of GluN3A subunit protein puncta (*orange arrowheads*) relative to PSD-95 (*white arrowheads*) and Bassoon (*yellow arrowheads*) at single synapses under high magnification. Note that GluN3A subunit puncta colocalize with PSD-95 and together paired with but well-separated from Bassoon, defining functional synapses.

Having established postsynaptic expression of the glycine binding GluN1 and GluN3A subunits independently, we sought to determine their colocalization with glutamate binding GluN2 subunits to make *t*-NMDARs. Given that we could only image four fluorophores at a time, we chose to immunoassay GluN1, GluN2A, and GluN3A subunit proteins along with MAP2, knowing that both GluN1 and GluN3A puncta coalesce precisely with the postsynaptic marker PSD-95. GluN1, GluN2A, and GluN3A subunit protein puncta in the MEA were imaged separately at various enlargements and merged to assay colocalization (*top two rows*, Figure 3A). Note that GluN1 and GluN3A puncta coalesce precisely with the GluN2 puncta, and this is better appreciated by altering the pseudocolor antigen key at higher magnifications (*arrowheads, bottom three rows*, Figure 3A). The *merge matrix* depicted in Figure 3B enables pairwise assessment of colocalization of the GluN1, GluN2A, and GluN3A subunit proteins at a single synapse on a dendrite at the highest level of magnification (note changes in color of the merged channels aid in assessing overlap). The merged images in the rightmost column of Figure 3B offer the first glimpses of the *t*-NMDAR subunit composition, comprising two glycine binding subunits (GluN1 and GluN3A) and two glutamate binding subunits (GluN2A) as described previously (Pilli and Kumar, 2012; Kumar, 2016; Beesley et al., 2019). To probe colocalization of these subunit proteins further, we delved deeper into the tissue moving from optical section 7 (Figures 3A, B) to 13 (Figures 4A, B; *inset*, *bottom right panel*, Figure 4B). As before, there was clear colocalization of the GluN1, GluN2A and GluN3A subunit protein puncta at various magnifications (*arrowheads*, *top two rows*, Figure 4A) and the high signal to noise ratio even permitted visualization of spinous protrusions (putative spine heads) emanating from the MAP2 labeled dendrites (*white arrowheads in bottom two rows*, Figure 4A) that seem to contain the sites (PSD, *demarcated by yellow arrowheads in third row from top*, Figure 4A) at which the three subunit proteins colocalize (*bottom two rows*, Figure 4A). The merge matrix shown in Figure 4B enables pairwise assessment of this colocalization at high magnification (note the change in pseudocolor antigen key).

To reconfirm their postsynaptic origin, we immunoassayed for GluN3A and GluN2A subunit proteins together with MAP2 and PSD-95 in a separate set of experiments looking into the MEA (Figure 5A) and hippocampus (Figure 5B). GluN3A and GluN2A puncta colocalize with PSD-95 at the level of the dendrites, as can be seen by correlating the merged images of GluN2A and GluN3A (*rightmost column*, Figure 5) with PSD-95 (*leftmost column*, Figure 5) at various magnifications (*top three rows*, *yellow arrowheads*, Figures 5A, B), although not all subunits colocalize with PSD-95 or with each other (*yellow circles*, Figure 5A). Interestingly, we found conspicuous dense immunolabeling of GluN2A and GluN3A subunit proteins, but not PSD-95, in the perikaryon of the cell bodies (*white arrowheads*, *rows 4 and 5*, Figure 5A), but not the nucleus, that attests to the specificity of the antibodies used and serves as an internal control for colocalization of subunit puncta with the postsynaptic marker. We found a similar pattern of immunolabeling in the hippocampus (Figure 5B). Note the precision with which the coalesced subunits align with the postsynaptic marker at the level of the dendrite under high magnification (*bottom row*, Figure 5B). Together, these data provide firsthand evidence of the coalescing of GluN1, GluN2A, and GluN3A subunit proteins for making *t*-NMDARs postsynaptically.

### GluN1 and GluN3 subunits do not colocalize with the presynaptic marker Bassoon

To rule out the possibility of presynaptic *t*-NMDAR expression, we immunoassayed separately for the glycine binding subunits GluN1 (mandatory for making NMDARs) and GluN3A, together with the dendritic marker MAP2 and Bassoon, one of two (the other being Piccolo) very large scaffolding proteins of the cytomatrix assembled at the active zone of excitatory and inhibitory synapses where neurotransmitter is released (Richter et al., 1999; Gundelfinger et al., 2015). We found extensive labeling of Bassoon throughout the MEA neuropil which stood out from the labeling for PSD-95 and/or GluN1 at various magnifications (Figure 6). Bassoon labeled puncta were restricted mostly to intradendritic spaces and paired, but not colocalized, with PSD-95 (*red circles*, *top row*, Figure 6A) and/or GluN1 (*yellow circles with embedded arrowheads*, Figures 6A–C). Note the distinction between colocalization of GluN1 puncta with PSD-95, and together, their pairing with a clearly separated Bassoon (Figures 6B, C). The separation between PSD-95 and Bassoon varied between pairings and likely depends on the orientation of the synapse within the optical plane. Not all GluN1/PSD-95 colocalizations could be associated with visible Bassoon puncta (*yellow circles*, Figures 6A–C) and conversely, not all Bassoon puncta were associated with PSD-95 and/or GluN1. To more closely examine the colocalization of GluN1/PSD-95 puncta and their separation from the presynaptic marker Bassoon, we looked at single synapses under high magnification (Figures 6D–F). Note the location of Bassoon (*yellow arrowheads*, *both rows*, Figure 6D) relative to PSD-95 (*white arrowheads*, *both rows*, Figure 6D) and GluN1 (*orange arrowheads*, *both rows*, Figure 6D), with or without the dendrite (MAP2) in the background (*top and bottom rows, respectively*, Figure 6D). The enlarged images (Figures 6E, F), with the pseudocolor antigen key altered to assess colocalization, clearly show that the presynaptic marker Bassoon is separated from the postsynaptic marker PSD-95, defining the synapse, and that colocalization of GluN1 is with PSD-95 but not Bassoon. We noted additionally that expression of the GluN1 puncta relative to PSD-95 (P) and Bassoon (B) might be indicative of synaptic orientation (*dotted lines with arrows*, Figures 6E, F) in the optical plane imaged. To confirm if GluN3A subunit protein follows the GluN1 pattern of expression, we immunoassayed for it together with the dendritic marker MAP2 and Bassoon (Figure 7). Like GluN1, GluN3A also colocalized with PSD-95 and both pair but do not coalesce with Bassoon independently or as a single unit at various magnifications (*top two rows*, *yellow circles*, Figure 7A). The high magnification images (Figure 7B) clearly show that the presynaptic marker Bassoon (*yellow arrowheads*, Figure 7B) is again separated from the postsynaptic marker PSD-95 (*white arrowheads*, Figure 7B) at the level of single synapses, and that colocalization of GluN3A (*orange arrowheads*, Figure 7B) is with PSD-95 but not Bassoon. Together, these data confirm the postsynaptic expression of GluN1 and GluN3 subunit proteins and by extension of *t*-NMDARs of which they are comprised.

### Control experiments

Even though agglomerations of subunit puncta with PSD-95 or among themselves cannot be attributed to chance given the number of colocalizations, we nonetheless performed three specific types of control experiments to legitimize these occurrences.

#### Antibody controls

To revalidate in house, the specificity of the commercially obtained *primary antibodies* against *t*-NMDAR subunits and to rule out any cross reactivity between them, we expressed GluN1, GluN2A, and GluN3A subunits individually in HEK 293 cells using fluorescent protein-tagged, subunit-specific plasmids (see cell *culture and transfection* in Methods) and assayed for them sequentially using the commercially obtained primary antibodies following visual verification of their expression (Supplementary Table 1; see *immunoblotting* in the *cell biology* section of Methods). Our results suggest that the antibodies recognize their respective antigens with high specificity and little to no cross reactivity (Supplementary Figure 1).

#### Histology controls

To rule out cross reactivity of fluorophore-conjugated *secondary antibodies* with incorrect primary antibodies and to gauge non-specific immunofluorescence. For this, we immunolabeled MAP2 with a rabbit primary (Supplementary Table 1) and a goat anti-rabbit biotin/streptavidin 647 secondary (Supplementary Table 2) to visualize dendrites, followed by immunolabeling of GluN1 with a guinea pig primary and incubations with a goat anti-guinea pig Alexa 555 secondary and an additional unconjugated goat anti-rabbit secondary (to saturate as many epitopes on the first primary as possible). We then assayed for cross immunofluorescence of the other fluorophore-conjugated secondary antibodies used in our protocols–goat anti-rabbit Alexa 594 and goat anti-rabbit Alexa 488 under high magnification. There was little to no cross immunofluorescence of the secondaries with the primaries, thereby validating their specificity and our approach for sequential immunolabeling of antigens (Supplementary Figure 2A; also see section on *immunofluorescence* in Methods). We reassessed this again by immunolabeling PSD-95 and GluN1 with rabbit and guinea pig primaries (Supplementary Table 1) and visualizing them with goat anti-rabbit Alexa 488 and goat anti-guinea pig Alexa 555 secondary antibodies, respectively (Supplementary Table 2), followed by incubation with an unconjugated goat anti-rabbit antibody. We then assayed for cross immunofluorescence of the goat anti-rabbit biotin-streptavidin 647 (secondary only) and goat anti-rabbit Alexa 594 secondary. As before, there was little to no cross immunofluorescence of the fluorophore-conjugated secondaries with the primaries (Supplementary Figure 2B).

#### Region and tissue-specific controls

NMDAR subunit controls were assayed in this study by evaluating their expression patterns in brain areas other than the MEA and hippocampus and in non-neuronal tissue. For region-specific controls, we assayed for the expression of GluN1, GluN2A, and GluN3A subunits in the medulla oblongata (Supplementary Figure 3) and the cerebellum (Farrant et al., 1994; Llansola et al., 2005; Supplementary Figure 4) and used liver tissue as our non-neuronal control (Supplementary Figure 5). There was sparse expression of all three subunits in both the medulla and cerebellum compared to either the MEA or hippocampus. In the cell-dense neuropil of the medulla, we found both somatic and dendritic expression of GluN1, GluN2A and GluN3A subunit proteins in punctate form (Supplementary Figures 3A, B) that coalesced occasionally to make putative *t*-NMDARs (Supplementary Figure 3C) and conventional GluN1/GluN2A-containing *d*-NMDARs (Supplementary Figure 3D). However, unlike MEA or hippocampus, we also observed for the first time, co-expression of GluN3A with GluN2A, but not GluN1, suggesting that the two subunits can come together as dimers (Supplementary Figure 3E). Whether these can dimerize further to make functional NMDARs is unknown. This pattern of expression was also observed in the cerebellum proper (Supplementary Figures 4A, B) with the expression of putative *d*- and *t*-NMDARs (Supplementary Figures 4C, D) and GluN2A/GluN3A dimers. As in the medulla, we estimated ∼50% of the dimers to be GluN2A/GluN3A expressing (Supplementary Figure 4E), 10% to be GluN1/GluN2A expressing and the rest as expressing all three subunits, given that we found few, if any, dimers containing just GluN1 and GluN3A. The expression of NMDAR subunits in the liver (non-neuronal control tissue) was even sparser than in the cerebellum or medulla, but not totally absent (Supplementary Figure 5). Furthermore, the MAP2 antibody which showed specificity for dendritic processes associated with neurons (as opposed to astroglia) in brain tissue, likely immunolabeled a variant of MAP2 protein in liver tissue revealing hepatocytes (confirmed using the nuclear DAPI stain; Supplementary Figure 5A). Expression of GluN1 within the cell bodies of hepatocytes and/or non-parenchymal liver cells was higher than the expression of GluN2A and GluN3A combined, and these subunit puncta could only be visualized properly under high magnifications (bottom row of images in Supplementary Figures 5A, B) given their sparse expression. We occasionally came across puncta containing the GluN1, GluN2A, and GluN3A subunit proteins needed for making *t*-NMDARs, although a large majority of the puncta were GluN2A/GluN3A dimers devoid of GluN1 (white arrow heads point to missing subunits; Supplementary Figure 5A, immunolabeled with MAP2). Many of these dimers were in the cytoplasm, judging from their expression relative to the well-demarcated nuclei (labeled with DAPI; Supplementary Figure 5B). Together, these data constitute regional and tissue-specific controls for the NMDAR subunit proteins assayed in the MEA and hippocampus.

## Discussion

Visualizing receptor subunit composition is essential for reconciling electrophysiological, cell biological and pharmacological data with function. This is especially true for NMDARs that are functionally very diverse. Studies of recombinant receptors have suggested that variations in subunit composition endow NMDARs with their functional diversity (Cull-Candy et al., 2001; Paoletti et al., 2013) although the precise makeup of native receptors and their expression patterns in the brain has remained largely unknown. The discovery and cloning of the GluN3 subunits (GluN3A-B), the final members of the NMDAR family, augmented the functional diversity of conventional glutamate-activated GluN1/GluN2(A-D) containing NMDARs by introducing unconventional glycine-activated GluN1/GluN3(A-B) NMDARs that were originally thought to form relatively Ca^2+^-impermeable cation channels and be expressed presynaptically (Das et al., 1998; Chatterton et al., 2002; Perez-Otano and Rodriguez-Moreno, 2019; Crawley et al., 2022). We showed previously that the GluN3 subunit can combine with GluN1 and GluN2 (A and/or B) to make glutamate activated *t*-NMDARs which are distinguishable from GluN2-containing *d*-NMDARs electrophysiologically (have excitatory postsynaptic currents with markedly different current-voltage relationships), have reduced affinity for Mg^2+^, and increased selectivity for Ca^2+^ over Na^+^, making them highly Ca^2+^ permeable (Pilli and Kumar, 2012, 2014; Beesley et al., 2019, 2020b; Kumar and Kumar, 2021). These receptors are blocked by the pan-NMDAR antagonist D-(-)-2-Amino-5-phosphonopentanoic acid (D-AP5) and by D-serine, a potential gliotransmitter and a co-agonist of conventional NMDARs (Kumar, 2016; Beesley et al., 2019, 2020a). Furthermore, we showed that NMDAR subunit composition can vary, not only between different types of neurons, but also between different synaptic inputs onto a neuron and even at a single synapse and that these differences are specific to NMDARs but not coexpressed α-amino-3-hydroxy-5-methyl-4-isoxazolepropionic acid receptors (AMPARs) (Kumar and Huguenard, 2003; Pilli and Kumar, 2012).

The present study is an attempt at visualizing *t*-NMDAR subunit composition at excitatory synaptic inputs onto pyramidal neurons in the medial entorhinal cortex, a hub of spatial navigation (Hafting et al., 2005) and memory consolidation, interfacing the hippocampus, where memories are initially formed, and the neocortex, where they are eventually rendered for permanent storage (Iijima et al., 1996; Tronson and Taylor, 2007). Our data provide direct evidence for colocalization of the two glycine binding subunit proteins, GluN1 and GluN3A, with glutamate binding GluN2A subunits for making synaptic *t*-NMDARs. Note that colocalization alone does not imply interaction and it is conceivable for these subunits to assemble separately as GluN1/GluN3A and GluN1/GluN2A *d*-NMDARs, but unlikely, because of the different neurotransmitters required for their activation and the perpetually desensitized state in which GluN1/GluN3A *d-*NMDARs would find themselves given the continuous albeit controlled availability of glycine at the synaptic cleft and/or be rendered permanently antagonized by ambient D-serine *in vivo* (Berger et al., 1998; Chatterton et al., 2002; Pilli and Kumar, 2012) c.f. excitatory glycine GluN1/GluN3A receptors (eGlyRs) (Grand et al., 2018; Otsu et al., 2019; Bossi et al., 2022). Moreover, PSD-95 binds specifically with the GluN2 subunit of the NMDARs and serves as a multidomain anchoring protein for many scaffolding and structural proteins postsynaptically (Sweatt, 2008; Stanic et al., 2015) thereby increasing the likelihood of its interaction with GluN3. The alternate possibility of *t*-NMDARs assembling with GluN1/GluN2A *d*-NMDARs at single synapses however, cannot be ruled out because both receptor types are activatable by glutamate and blocking synaptic *t*-NMDARs in the MEA pharmacologically has been shown to unmask *d*-NMDARs, with all responses being antagonized by D-AP5 (Beesley et al., 2019). Colocalization of functionally distinct NMDAR subtypes at individual synaptic inputs likely enhances the repertoire of neurons for information processing and plasticity within the entorhinal cortex (Pilli and Kumar, 2014). Our data also establish the postsynaptic locus of expression of these receptors by examining the colocalization of various subunits with PSD-95 but not with the presynaptic marker Bassoon. Although we could not resolve finer details of region and/or lamina specific information owing to the high magnifications used for visualization, we did not observe subunit puncta to colocalize individually or as a cohort with Bassoon, thereby ruling out presynaptic expression of these receptors within the MEA and/or CA1-3 hippocampus.

An upshot of this work is the possibility of visually analyzing pathology underlying neurodegenerative disorders like temporal lobe epilepsy (TLE) from the synaptic/receptor perspective. For example, we have previously shown how *t*-NMDARs, by virtue of their increased selectivity for Ca^2+^ render neurons vulnerable to excitotoxic damage and contribute to the pathology (vulnerability and pattern of neuronal loss) and by extension to the pathophysiology (Ca^2+^-induced excitotoxicity) underlying TLE (Beesley et al., 2020a; Kumar and Kumar, 2021). By assaying the spatial expression of their subunit proteins (GluN1, GluN2A, GluN2B, and GluN3A) using area-specific tissue analysis (ASTA), a novel methodology for harvesting brain chads from hard-to-reach regions within brain slices for Western blotting, we recently showed that GluN3A was expressed in a gradient along the mid-lateral extent of layer three MEA and along the CA1-subicular axis in the hippocampus, unlike GluN1 and GluN2A which were uniformly distributed. The expression profile of GluN3A defined the “zones of vulnerability” in these regions where there was significant cell loss and neurodegeneration, hallmark features of the disease (Beesley et al., 2022). Thus, the GluN3A expression pattern was indicative of the spatial extent of the pathology in the hippocampus and entorhinal cortex implicating *t*-NMDARs in TLE pathogenesis. Future studies will be able to use the methodology described here to examine spatiotemporal changes in the expression patterns of specific NMDAR subunit proteins visually as a function of disease progression by incorporating data from epileptic animal models to better characterize TLE pathology.

The paucity of subunit-specific compounds has been the bane of NMDAR research until recently, especially for triheteromeric receptors, hindering characterization of their biophysical and functional properties and assessments of their expression and role in the brain. This has even stymied progress on seeking molecules with which to pursue therapeutic options for a wide variety of diseases that implicate them (Paoletti and Neyton, 2007; Stroebel et al., 2018). Furthermore, many of the compounds available for NMDAR subunit pharmacology are not specific enough and there is a niche for alternative approaches to assess subunit composition. The present study, undertaken in the spirit of seeing is believing, is aimed at fulfilling this niche through direct visualization of subunit composition using subunit-specific antibodies and high-resolution confocal microscopy as described in this work.

## Data availability statement

The original contributions presented in this study are included in the article/ Supplementary material, further inquiries can be directed to the corresponding author.

## Ethics statement

The animal study was reviewed and approved by Florida State University Institutional Animal Care Committee.

## Author contributions

SK and SB designed and analyzed all experiments outlined in this manuscript. SB contributed to all the cell biology, histology, and confocal microscopy. AG contributed to transfections of HEK cells with plasmids for heterologous expression of GluN subunits to validate the specificity of the antibodies used in this work and provided guidance with microscopy. SK contributed to the electrophysiological assessment of *t*-NMDAR function and writing the manuscript. All authors contributed to the article and approved the submitted version.
