# Supplementary material for: Visualizing the triheteromeric N-methyl-D-aspartate receptor subunit composition
Source: Front Synaptic Neurosci. 2023 May 24;15:1156777. doi: 10.3389/fnsyn.2023.1156777 (PMC10244591; doi:10.3389/fnsyn.2023.1156777)
Supplement: Supplementary file 1 [file Data_Sheet_1.pdf]

Supplementary Table 1

primary antibody	source	reference #	lot	species	concentration (original)	dilution used
GluN1	Alomone Labs	AGP-046	AGP046AN0150	Guinea Pig	N/A	1:500
GluN2a	Alomone Labs	AGC-002	AGC002AN0650	Rabbit	N/A	1:500
GluN3a	Alomone Labs	AGC-030	AGC030AN0250	Rabbit	N/A	1:500
MAP2	Abcam	Ab32454	GR3281998-1	Rabbit	1 mg/ml	1:500
Bassoon	Enzo	ADI-VAM-PS003-D	02012005	Mouse	1 mg/ml	1:500
PSD-95	Invitrogen	51-6900	VH307495	Rabbit	0.25 mg/ml	1:500

Supplementary Table 1: Details of the primary antibodies used for immunolabeling.

Supplementary Table 2

**secondary antibody**	**source**	**reference #**	**lot**	**concentration** (original)	**dilution used**
Goat anti-rabbit Alexa 488	Invitrogen	A11008	2179202	2 mg/ml	1:500
Goat anti-rabbit Alexa 555	Invitrogen	A21429	2298171	2 mg/ml	1:500
Goat anti-rabbit Alexa 594	Invitrogen	A11037	2160403	2 mg/ml	1:500
Goat anti-guinea pig Alexa 555	Invitrogen	A21435	2373245	2 mg/ml	1:500
Goat anti-guinea pig Alexa 594	Invitrogen	A11076	2409040	2 mg/ml	1:500
Goat anti-mouse Alexa 488	Invitrogen	A11001	1787787	2 mg/ml	1:500
Goat anti-rabbit Biotin	Invitrogen	A16114	81-82-051721	1.5 mg/ml	1:500
Streptavidin 647	Invitrogen	S32357	1932748	2 mg/ml	1:500
Goat anti-rabbit unconjugated	Invitrogen	31210	VK3130312	2.32 mg/ml	1:500

Supplementary Table 2: Details of the secondary antibodies used for immunolabeling.

Supplementary Fig 1: **Validation of the primary antibodies used in *t*-NMDAR immunohistochemistry/imaging through Western blotting.**

a-c, To determine in house the specificity of the commercially obtained primary antibodies used in this study and to confirm any cross-reactivity, we overexpressed each of the three GluN subunits, GluN3A (**a**), GluN1 (**b**) and GluN2A (**c**) separately in HEK 293 cells (see *cell culture and transfection*, in Methods for details of the plasmids used for transfection). Cytoplasmic fractions were extracted and immunoblotted against the primary antibodies used for immunofluorescence (supplementary Table 1). Incubation with the anti-GluN3A antibody showed a clear band at the expected molecular weight with no cross reactivity with either GluN1 or GluN2A, suggesting antigen specificity (band boxed with a red hatched line overlayed on the image of the actual gel used for the experiment, **a**). We noted similar results following sequential incubations with the anti-GluN1 antibody (no cross reactivity with either GluN3A or GluN2A, **b**), and the anti-GluN2A antibody (no cross reactivity with either GluN3A or GluN1, **c**), thereby confirming their specificity.

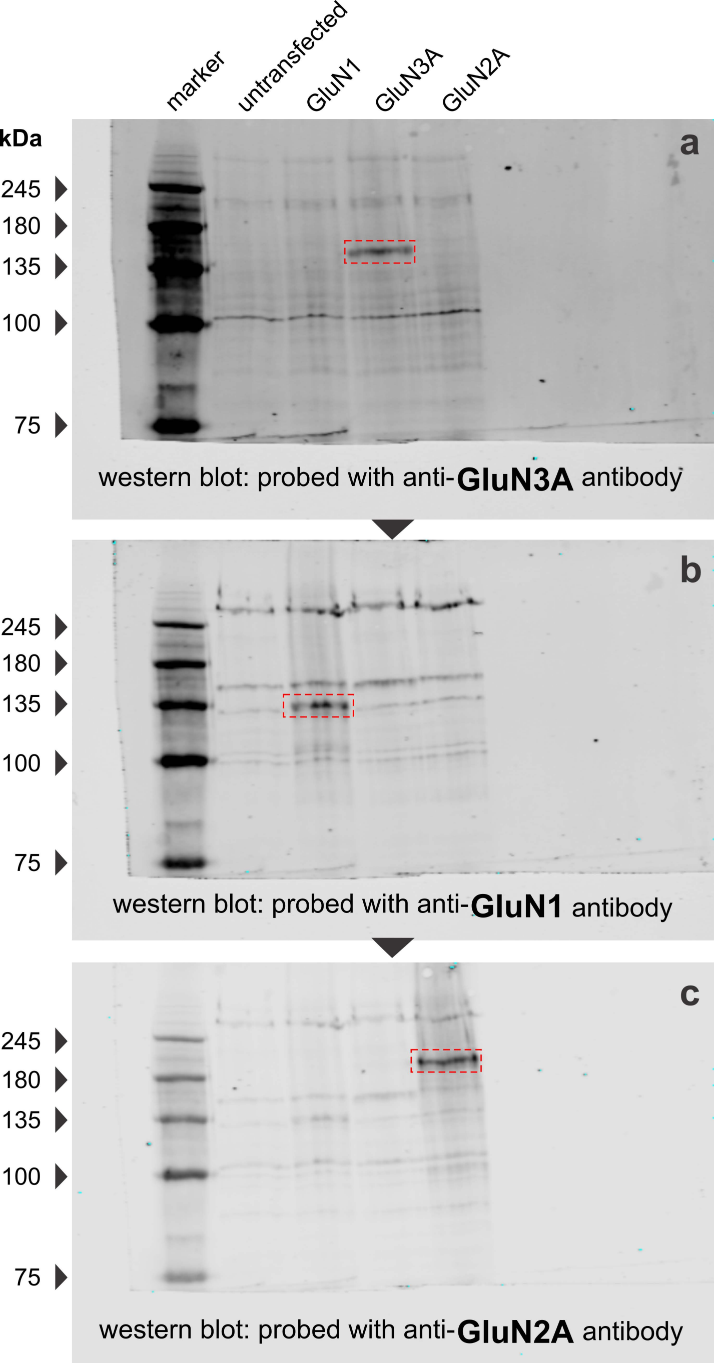
marker
untransfected
GluN1
GluN3A
GluN2A
kDa
a
245
180
135
100
75
western blot: probed with anti-GluN3A antibody
b
245
180
135
100
75
western blot: probed with anti-GluN1 antibody
c
245
180
135
100
75
western blot: probed with anti-GluN2A antibody

Supplementary Fig 2: **Validation of the secondary antibodies used in *t*-NMDAR immunohistochemistry/imaging.**

a-b, To rule out cross reactivity between the secondary (S) antibodies used in our immunofluorescence work, given that many of primary (P) antibodies used had rabbit as their host species (supplementary Table 1), we used anti-rabbit secondary only controls in conjunction with a single rabbit primary and an anti-rabbit unconjugated antibody (see *immunofluorescence*, in Methods for specific details) to demonstrate that the several anti-rabbit secondary antibodies used (test-1 and test-2) do not bind to the same primary, thereby confounding results (schematically shown atop **a** and **b**). **a**, Slices were incubated overnight with anti-MAP2 (P: rabbit host) and anti-GluN1 (P: guinea pig host) prior to being immunolabelled with anti-rabbit biotin/streptavidin-647(S) and anti-guinea pig Alexa-555 (S1). The slices were incubated with an unconjugated goat anti-rabbit secondary antibody (S2) before being exposed to goat anti-rabbit Alexa-594 (test-1) and goat anti-rabbit Alexa-488 (test-2). Panel of images in the bottom row (B) are enlargements of the boxed area indicated in yellow in the leftmost image on the top row (A). **b**, Likewise, slices were incubated overnight with anti-PSD-95 (P: rabbit host) and anti-GluN1 (P: guinea pig host) prior to being labelled with anti-rabbit Alexa-488 (S1) and anti-guinea pig Alexa-555 (S1) secondary antibodies respectively (supplementary Table 2). The slices were incubated with an unconjugated goat anti-rabbit secondary antibody (supplementary Table2) before incubation with anti-rabbit biotin followed by streptavidin-647 (test-1) and anti-rabbit Alexa-594 (test-2). Panel of images in the bottom row (B) are enlargements of the boxed area indicated in yellow (a single synapse) in the leftmost image on the top row (A). Note absence of cross immunofluorescence from the fluorophore-conjugated test antibodies in the merged images (*rightmost column* of images in **a** and **b**).

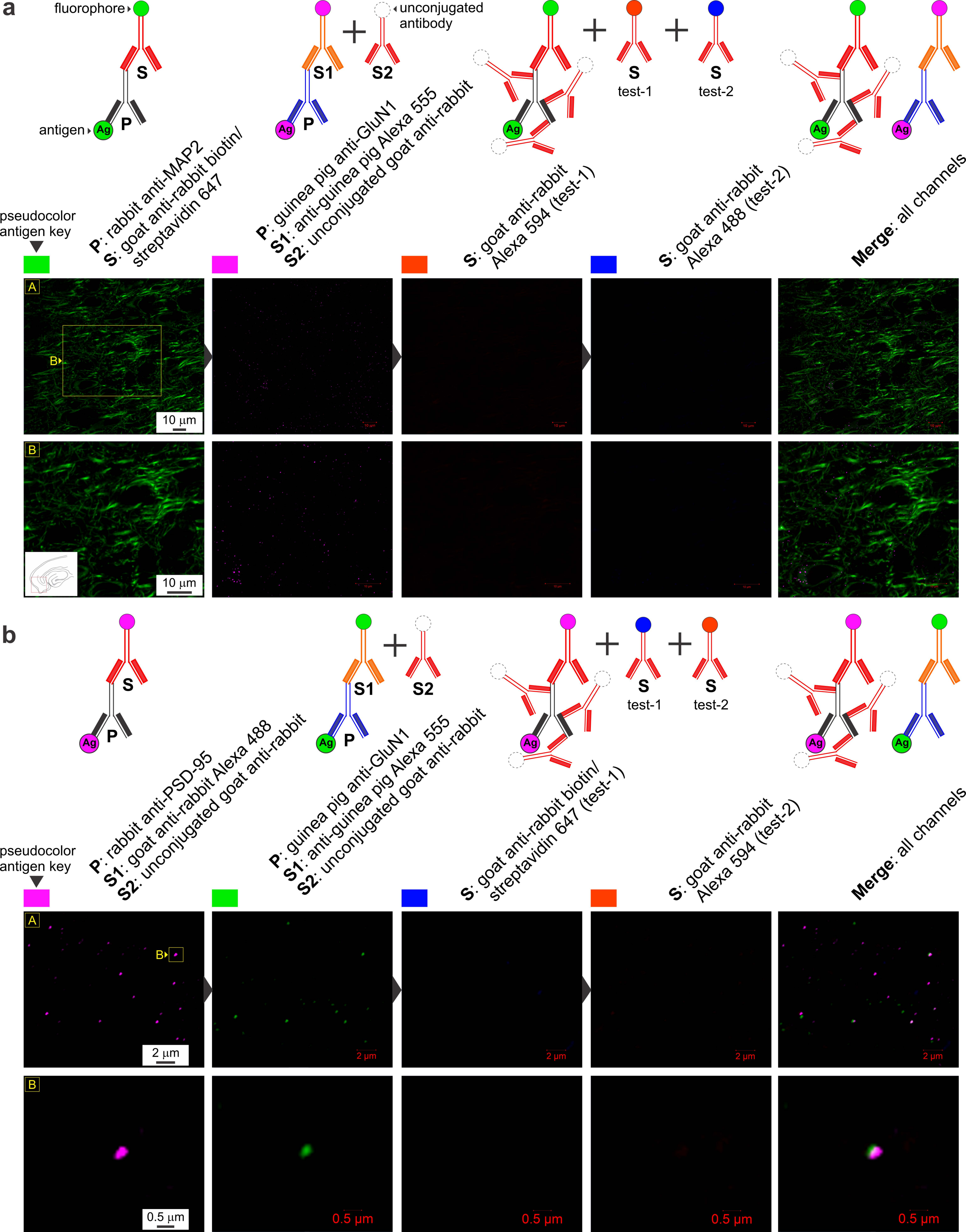

a
fluorophore
antigen
Ag
P
S
S1
S2
unconjugated antibody
test-1
test-2
pseudocolor antigen key
P: rabbit anti-MAP2
S: goat anti-rabbit biotin/ streptavidin 647
P: guinea pig anti-GluN1
S1: anti-guinea pig Alexa 555
S2: unconjugated goat anti-rabbit
S: goat anti-rabbit Alexa 594 (test-1)
S: goat anti-rabbit Alexa 488 (test-2)
Merge: all channels
A
B
10 µm
b
P: rabbit anti-PSD-95
S1: goat anti-rabbit Alexa 488
S2: unconjugated goat anti-rabbit
P: guinea pig anti-GluN1
S1: anti-guinea pig Alexa 555
S2: unconjugated goat anti-rabbit
S: goat anti-rabbit biotin/ streptavidin 647 (test-1)
S: goat anti-rabbit Alexa 594 (test-2)
Merge: all channels
2 µm
0.5 µm

Supplementary Fig 3: **Assaying expression of NMDAR subunit proteins in the medulla oblongata.**

a, Quadruple immunolabeling GluN1, GluN2A and GluN3A subunit puncta in the medulla oblongata (red/yellow boxes indicate region/area sampled in the medulla; R1C1) relative to dendrites immunolabeled with MAP2 (R1-2C2) at the indicated enlargements (lettered boxes in yellow). In this and subsequent figures, the *pseudocolor antigen key* indicates the color assignment for the antigens/fluorophores imaged/depicted and changes to the key (marked by || between images) are to aid in gauging colocalization of the proteins imaged.

b-e, Pairwise assessment of GluN1, GluN2A and GluN3A subunit protein colocalization at synapses at low (**b**) and ultrahigh (**c-e**) magnifications. The merged images in the rightmost column (R1-8C4) showcase the coalescing of glycine binding subunit proteins (GluN1 and GluN3A) with the glutamate binding subunit (GluN2A) to make putative *t*-NMDARs (**c**; inset in R2C4), conventional GluN1/GluN2A containing *d*-NMDARs (**d**; inset R2C4) and GluN2A/GluN3A dimers devoid of GluN1 (**e**; inset R2C4). Arrowheads point to colocalizing subunits (*yellow*) / missing (*white*) subunit puncta.

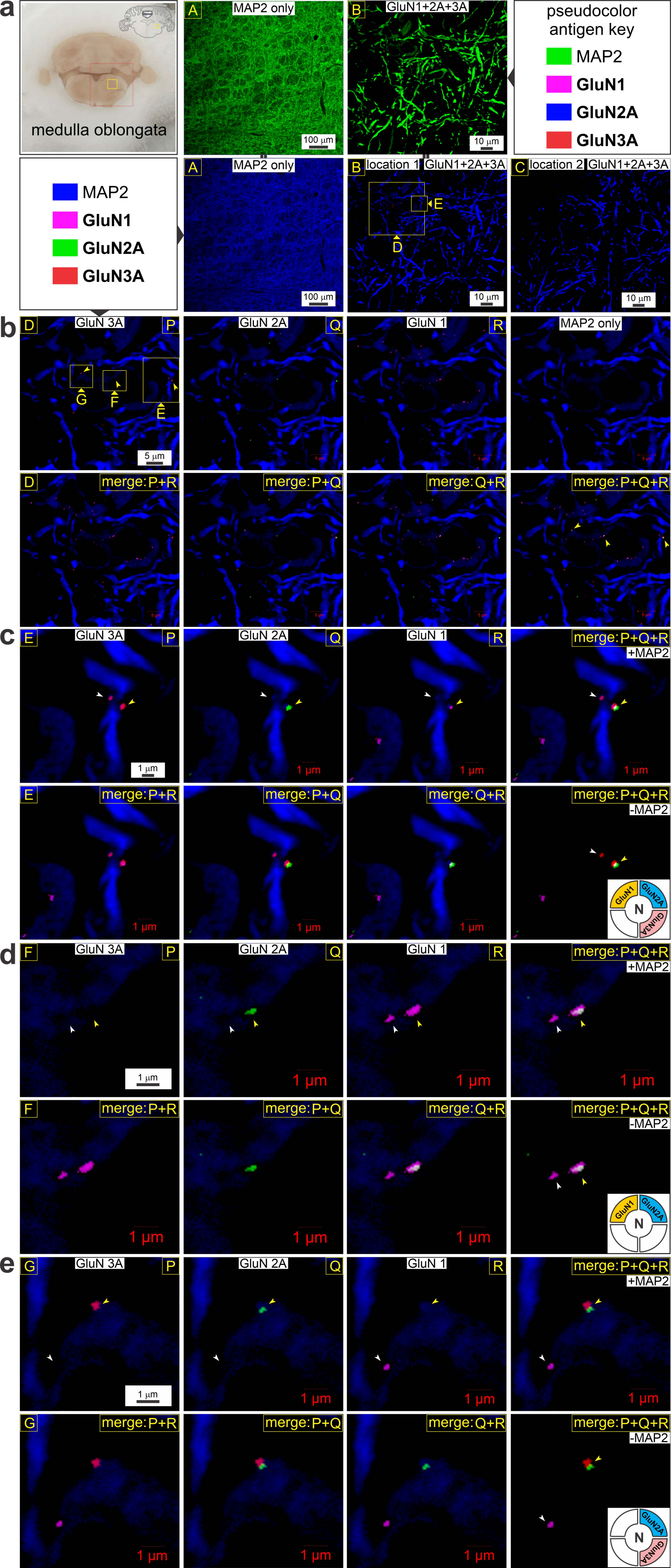

a
medulla oblongata
A MAP2 only
100 µm
B GluN1+2A+3A
10 µm
pseudocolor antigen key
MAP2
GluN1
GluN2A
GluN3A
MAP2
GluN1
GluN2A
GluN3A
A MAP2 only
100 µm
B location 1 GluN1+2A+3A
E
D
10 µm
C location 2 GluN1+2A+3A
10 µm
b
D GluN 3A P
G
F
E
5 µm
GluN 2A Q
GluN 1 R
MAP2 only
D merge: P+R
merge: P+Q
merge: Q+R
merge: P+Q+R
c
E GluN 3A P
1 µm
GluN 2A Q
1 µm
GluN 1 R
1 µm
merge: P+Q+R +MAP2
1 µm
E merge: P+R
1 µm
merge: P+Q
1 µm
merge: Q+R
1 µm
merge: P+Q+R -MAP2
GluN1
GluN2A
N
GluN3A
d
F GluN 3A P
1 µm
GluN 2A Q
1 µm
GluN 1 R
1 µm
merge: P+Q+R +MAP2
1 µm
F merge: P+R
1 µm
merge: P+Q
1 µm
merge: Q+R
1 µm
merge: P+Q+R -MAP2
GluN1
GluN2A
N
e
G GluN 3A P
1 µm
GluN 2A Q
1 µm
GluN 1 R
1 µm
merge: P+Q+R +MAP2
1 µm
G merge: P+R
1 µm
merge: P+Q
1 µm
merge: Q+R
1 µm
merge: P+Q+R -MAP2
GluN2A
N
GluN3A

Supplementary Fig 4: **Assaying expression of NMDAR subunit proteins in the cerebellum.**

a, Quadruple immunolabeling GluN1, GluN2A and GluN3A subunit puncta in the cerebellum proper (red/yellow boxes indicate region/area sampled in the cerebellum; R1C1-2) relative to MAP2 immunolabeling (R1C3-4) at the indicated enlargements (lettered boxes in yellow). The *pseudocolor antigen key* indicates the color assignment for the antigens/fluorophores imaged/depicted and changes to the key (marked by || between images) aid in gauging colocalization of the proteins imaged.

b-e, Pairwise assessment of GluN1, GluN2A and GluN3A subunit protein colocalization at synapses at low (**b**) and ultrahigh (**c-e**) magnifications. The merged images in the rightmost column (R1-8C7) showcase the coalescing of glycine binding subunit proteins (GluN1 and GluN3A) with the glutamate binding subunit (GluN2A) to make putative *d*-NMDARs containing GluN1/GluN2A (**c**; inset in R2C7), *t*-NMDARs containing GluN1/GluN2A/GluN3A (**d**; inset R2C7) and GluN2A/GluN3A dimers devoid of GluN1 (**e**; inset R2C7). Arrowheads point to colocalizing subunits (*yellow*) / missing (*white*) subunit puncta.

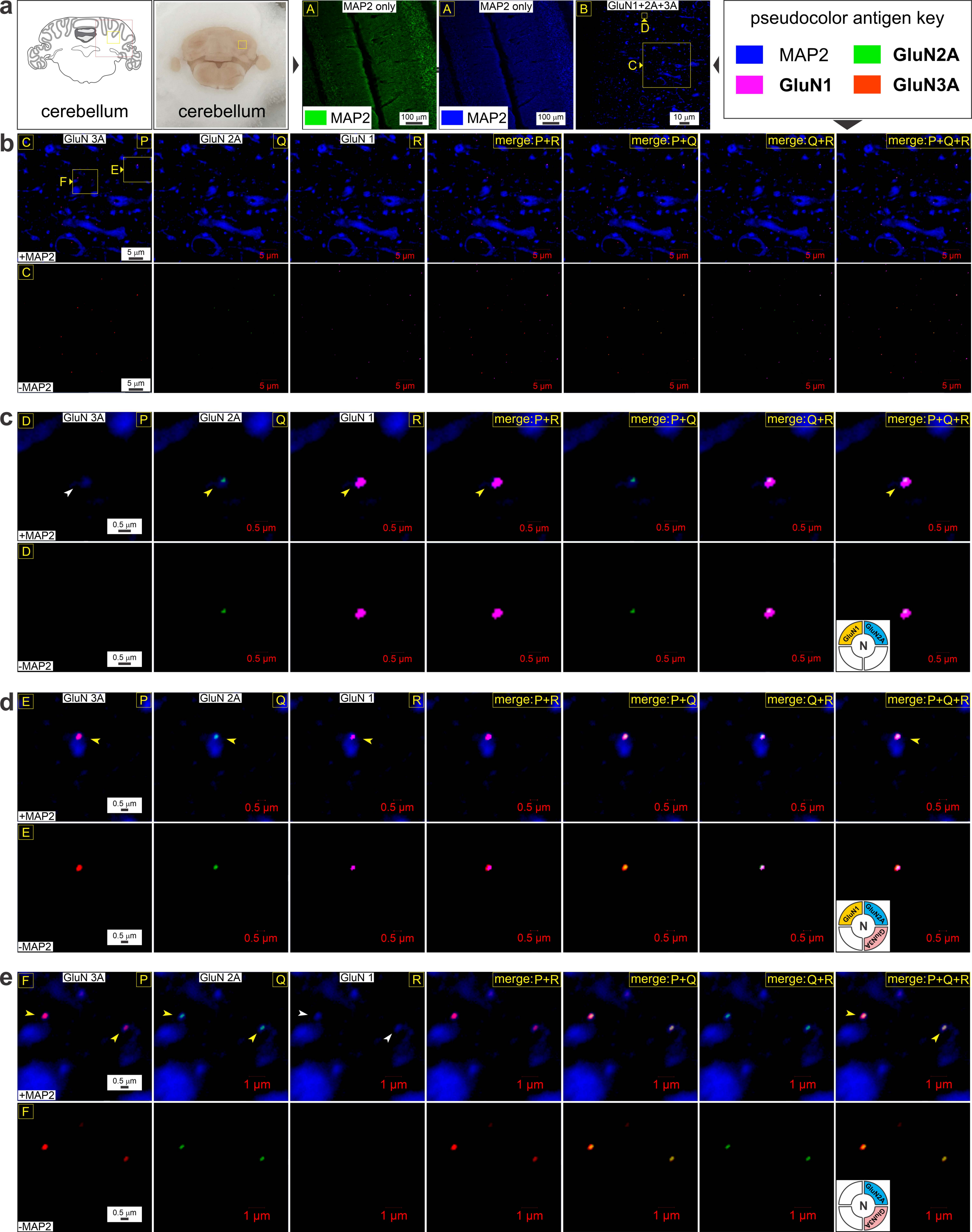

a
cerebellum
cerebellum
A MAP2 only
MAP2
100 µm
A MAP2 only
MAP2
100 µm
B GluN1+2A+3A
D
C
10 µm
pseudocolor antigen key
MAP2
GluN2A
GluN1
GluN3A
b
C GluN 3A P
E
F
+MAP2
5 µm
GluN 2A Q
GluN 1 R
merge:P+R
merge:P+Q
merge:Q+R
merge:P+Q+R
-MAP2
c
D GluN 3A P
GluN 2A Q
GluN 1 R
merge:P+R
merge:P+Q
merge:Q+R
merge:P+Q+R
0.5 µm
+MAP2
-MAP2
GluN1
GluN2A
N
d
E GluN 3A P
GluN 2A Q
GluN 1 R
merge:P+R
merge:P+Q
merge:Q+R
merge:P+Q+R
0.5 µm
+MAP2
-MAP2
GluN1
GluN2A
N
GluN3A
e
F GluN 3A P
GluN 2A Q
GluN 1 R
merge:P+R
merge:P+Q
merge:Q+R
merge:P+Q+R
0.5 µm
1 µm
+MAP2
-MAP2
GluN2A
N
GluN3A

Supplementary Fig 5: **Assaying expression of NMDAR subunit proteins in liver tissue.**

a, Immunolabeling GluN1, GluN2A and GluN3A subunit puncta in the liver (R1C1) relative to immunolabeling of a variant of MAP2 (R1C2-5) at the various enlargements (lettered boxes in yellow). DAPI in the mounting media labels nuclei of hepatocytes (R1C7). The *pseudocolor antigen key* indicates the color assignment for the antigens/fluorophores imaged/depicted and changes to the key (marked by || between images) aid in gauging colocalization of the proteins imaged. Note the sporadic expression of *t*-NMDARs and preponderance of GluN2/GluN3A dimers (insets, R5C7).

b, Pairwise assessment of GluN1, GluN2A and GluN3A subunit protein colocalization in hepatocytes (demarcated with DAPI) at low (R1-R2) and ultrahigh (R3-R6) magnifications. The merged images in the rightmost column (R1-6C7) showcase the coalescing of the glutamate binding GluN2A subunit with glycine binding GluN3A subunit to make GluN2A/GluN3A dimers devoid of GluN1 (**e**; inset R2C7). Arrowheads point to colocalizing subunits (*yellow*) / missing (*white*) subunit puncta.

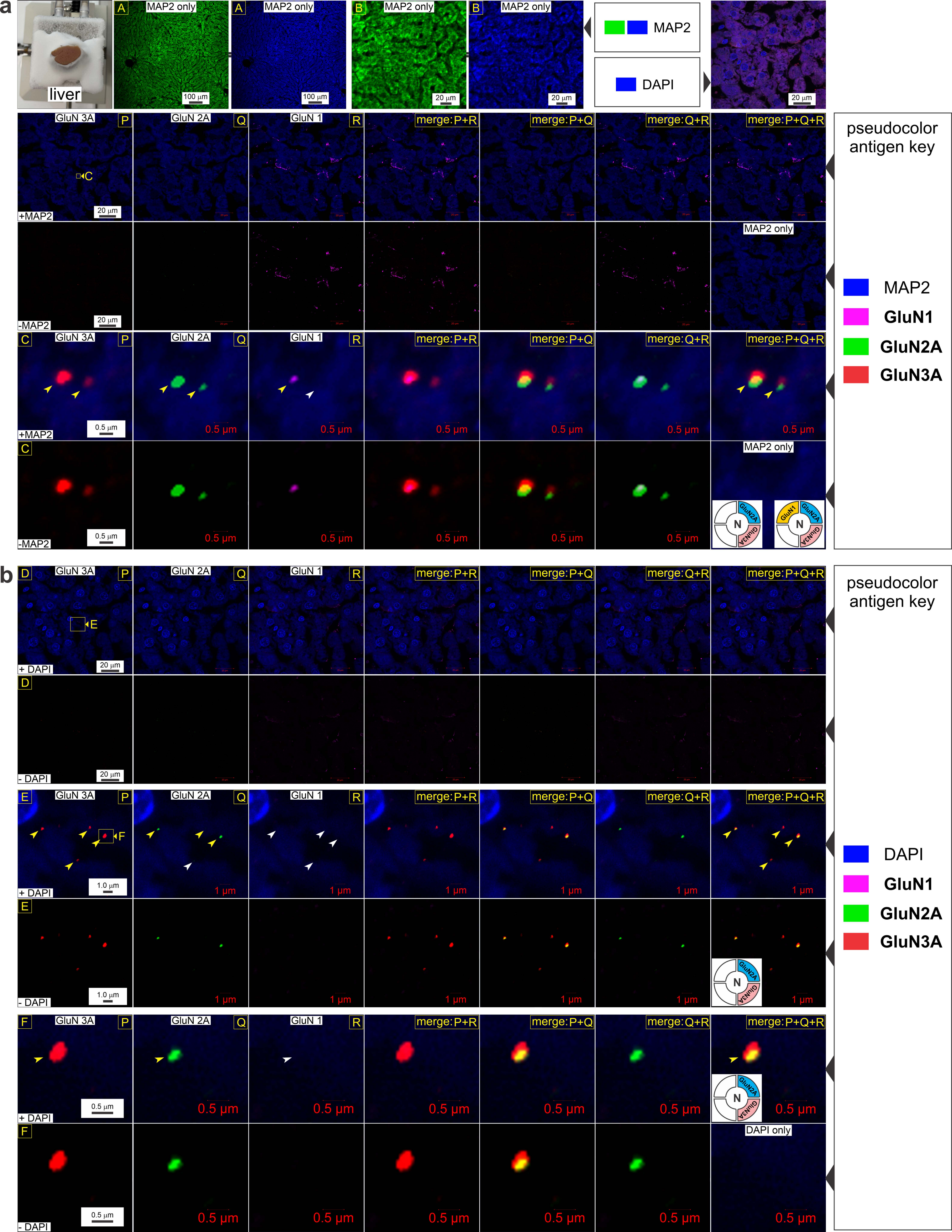

a
liver
A MAP2 only
100 µm
B MAP2 only
20 µm
MAP2
DAPI
GluN 3A P
GluN 2A Q
GluN 1 R
merge: P+R
merge: P+Q
merge: Q+R
merge: P+Q+R
C
+MAP2
-MAP2
MAP2 only
0.5 µm
GluN2A
GluN3A
GluN1
N
pseudocolor antigen key
MAP2
GluN1
GluN2A
GluN3A
b
D
E
F
+ DAPI
- DAPI
DAPI only
1.0 µm
1 µm
pseudocolor antigen key
DAPI
GluN1
GluN2A
GluN3A

Supplementary Fig 6: **An example showing how the viewing of confocal images can be optimized to reveal PSD-95 and NMDAR subunit puncta to study their colocalization.**

Screenshots of confocally acquired images processed using Zen 2012 SP1 (black edition; Carl Zeiss) software for visualizing at low (**a-b**) and high magnifications (**c**) immunolabeled PSD-95, GluN3A subunit puncta in the MEA relative to immunolabeled dendrites and nuclei using MAP2 antibodies and DAPI respectively. Raw images obtained from the confocal microscope are adjusted for saturation/exposure for each of fluorophore channels imaged using the range indicator function in the program (**a**, R1-R2). The raw images that are not yet optimized for viewing (**b**, R1-R2), are manually corrected for luminescence/contrast using Gamma (γ) /Black (B) /White (W) correction parameters for each channel separately based on the program's built-in Best Fit (**b**, R3) and Min/Max (**b**, R4) functions to yield the final images depicted in figure-form throughout the manuscript (panels boxed in *red*; **b**, R5; **c**, R3). Note that apart from the γ/B/W corrections, no other extraneous filtering is used to declutter the background noise (**b**, R3-R4, C1-C2; **c**, R1-R2, C1-C2) in each channel to optimize viewing of the brightest puncta for subunit expression (**b**, R5C1-2; **c**, R3C1-C2) and colocalization (**b**, R5C3-4; **c**, R3C3-C4) under the indicated magnifications.

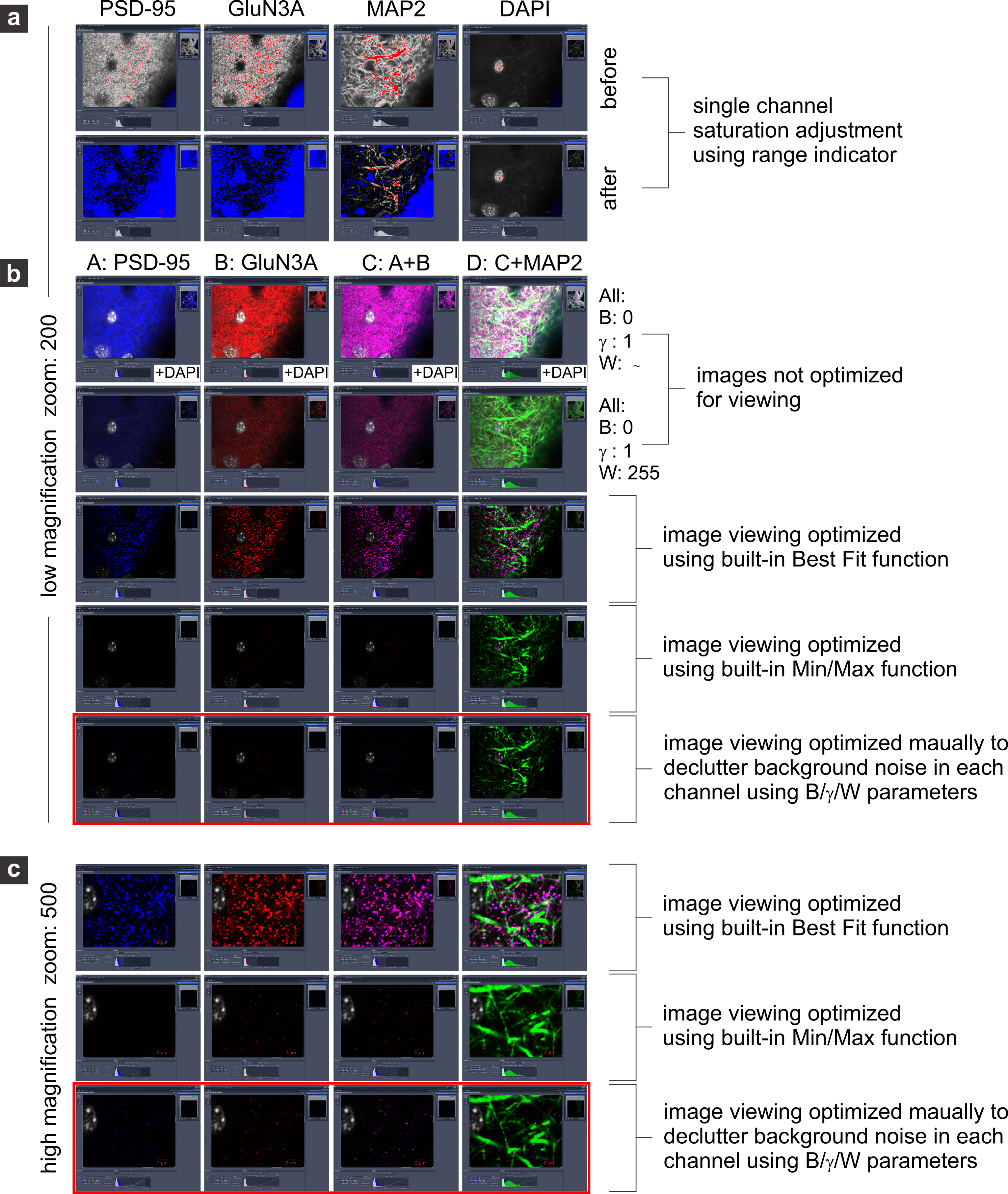
a
PSD-95
GluN3A
MAP2
DAPI
before
after
single channel
saturation adjustment
using range indicator
b
A: PSD-95
B: GluN3A
C: A+B
D: C+MAP2
+DAPI
All:
B: 0
γ : 1
W: ~
All:
B: 0
γ : 1
W: 255
images not optimized
for viewing
image viewing optimized
using built-in Best Fit function
image viewing optimized
using built-in Min/Max function
image viewing optimized maually to
declutter background noise in each
channel using B/γ/W parameters
low magnification zoom: 200
c
high magnification zoom: 500
image viewing optimized
using built-in Best Fit function
image viewing optimized
using built-in Min/Max function
image viewing optimized maually to
declutter background noise in each
channel using B/γ/W parameters
